# High-throughput system metabolomics method reveals new mechanistic insights of chlorogenic acid by using liquid chromatography coupled to high resolution mass spectrometry

**DOI:** 10.1039/c7ra12995e

**Published:** 2018-02-14

**Authors:** Yu Zhang, Yunxia Xiu, Chunna Ren, Cui Chen

**Affiliations:** Second Affiliated Hospital, Mudanjiang Medical University Mudanjiang Heilongjiang 157009 P. R. China tt_hljzyy@163.com +86 13694670225

## Abstract

It has increasingly been recognized that metabolism is highly interconnected with disease, and system metabolomics studies have aimed to discover metabolic biomarkers and analyze the pathways of metabolome phenotypes. To better understand the metabolic alteration related with disease, a urine metabolic profile using a high-throughput system metabolomics technology approach was applied to probe the underlying molecular mechanisms of alcohol-induced liver injury and the therapeutic effects of chlorogenic acid (CA). In this study, endogenous low-molecular-weight metabolites were characterized using liquid chromatography coupled with mass spectrometry (LC-MS). The acquired data was parsed by principal component analysis (PCA) and orthogonal partial least squares discriminant analysis (OPLS-DA) to identify potential biomarkers. A total of 19 biomarkers were identified in a model of alcohol-induced liver injury rats, and it was found that chlorogenic acid had a regulatory effect on 14 of them, associated with multiple metabolic pathways. Comprehensive pathway analysis suggests that CA has the ability to regulate abnormal metabolic states. In addition, accessory examinations such as biochemical analysis and histopathological observations were also performed that showed similar results. As a natural product agent against ethanol-induced liver injury, CA was validated in the rebalancing of a wide range of metabolic disorders. High-throughput system metabolomics represents a powerful approach for revealing new mechanistic insights of natural products.

## Abstract 2

It has increasingly been recognized that metabolism is highly interconnected with disease, and system metabolomics studies have aimed to discover metabolic biomarkers and analyze the pathways of metabolome phenotypes.

## Graphical abstract

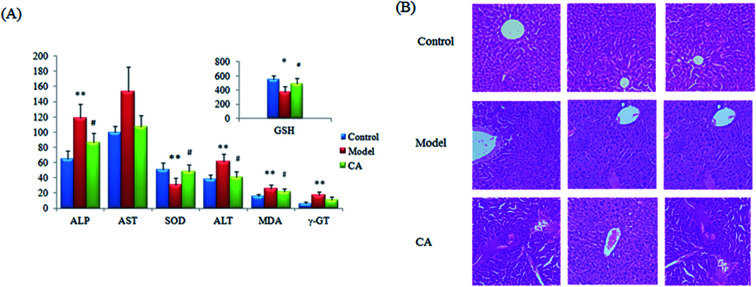

## 1. Introduction

As a part of systems biology, metabolomics is an integral method for the assessment of small molecules such as fatty acids, amino acids, peptides, nucleic acids, organic acids, vitamins, and carbohydrates in organisms or biological samples that refers to the qualitative and quantitative analysis of the overall biological sample metabolites.^1,2^ Metabolome presents actual internal environment condition in individuals, which was closely associated with external disturbance and internal fluctuations. Changes in the metabolite levels in an organism can be considered as the ultimate outcome of a combination of effects including environmental impact, genetic variance, microbes or enzyme kinetic activity changes, amongst others.^3,4^ Currently, there are not as yet efficient solutions for preventing or treating a great number of different diseases. The reasons for this situation may include measurements being performed too late or too early in the course of the disease, inadequate treatment targets on account of a lack in pathogenic knowledge and insufficient knowledge on the specific clinical features of the diseases.

Alcohol metabolism is hazardous to health due to a series of poisonous substances that arise during its progression that are difficult to eliminate. As a worldwide burden, alcoholic liver disease (ALD) represents the greatest impact that alcohol can have on the body, compared with any other diseases it causes, and refers to a serious health problem of excessive alcohol consumption, leading to chronic disease and even mortality. As we all know, ALD mainly relates to the disorder of a metabolic pathway, but traditional medical measurement methods such as pathological tests, biochemistry indexes and symptom assessments are unable to provide deep insight into pathological or metabolic alterations giving rise to liver injury that have been specifically induced by alcohol intake. Routine clinical examinations of the liver function in accordance with serum indexes, such as alanine amino-transferase (ALT) and aspartate aminotransferase (AST), are not sensitive and also fail to achieve early diagnosis.^5,6^ The metabolites in chaos during the ALD pathogenetic process enter the urinary environment and are then secreted in the urine. For the reasons given above, urine is a perfect source for selecting bio-samples in order to characterize the physiological environment in a non-invasive way. With a major improvement in modern metabolomics technology, it provides us with the opportunity to measure metabolites and obtain the metabolic fingerprint of individual patients in practice.^7^ These potential metabolites were deemed as diagnostic or prognostic tools that can be used to identify impairments in system occurring during the development and worsening of disease and assist clinicians to direct the stages differentiation and alter appropriate treatment.^8–10^

Compared with current available therapeutic options, natural products are becoming popular all over the world and are widely accepted as a conservative clinical therapy in the field of health promotion and disease treatment due to their effectiveness, convenience, fewer side-effects and relatively low cost.^11,12^ CA as a vital biosynthetic intermediate is the key ester component of caffeic acid and quinic acid,^13,14^ and has been discovered in a number of dietary plants and medicinal herbs such as *Phyllostachys edulis*^15^ and possesses strong anti-inflammatory,^16^ antioxidative,^17^ antihypertensive,^18^ antitumor,^19^ analgesic, antipyretic^20^ and other properties.^21^ Some previous studies have reported that CA reduces carbon tetrachloride-induced liver fibrosis and inhibits oxidative stress in liver and hepatic stellate cells.^22,23^ However, the mechanism of CA towards liver injury is still not well known. It is urgently needed to further research. Using an untargeted metabolomics method combined with a multivariate data method and pathway analysis, the aim of this study is to explore the potential mechanisms of metabolic disorder caused by ethanol in rats and the protective effect of CA using the profiling of urinary metabolites.

## 2. Materials and methods

### 2.1 Materials and reagents

Methanol (HPLC grade) was obtained from Fisher Scientific Corporation (Loughborough, UK); acetonitrile (ACN) (HPLC grade) was purchased from Merck (Darmstadt, Germany); formic acid was purchased from Honeywell Company (Morristown, New Jersey, USA); deionized water was produced by using a Milli-Q water ultra-pure water system (Millipore, Bedford, MA, USA); leucine enkephalin was purchased from Sigma-Aldrich (St. Louis, MO, USA). Chlorogenic acid (99% purity) was purchased from the Tianjin Chemical Reagent Co. (Tianjin, China). The assay kits for ALT, AST, alkaline phosphatase (ALP) and γ-glutamyl transpeptidase (γ-GT) were purchased from Biosino Company (Beijing, China). The assay kits for glutathione-Px (GSH-Px), maleicdialdehyde (MDA) and superoxide dismutase (SOD) were purchased from Nanjing Jiancheng Biological Research Institute (Nanjing, China). All other reagents were of analytical grade.

### 2.2 Animal models and treatment

Wister rats (weighing 200 ± 20 g, male) were purchased from the Shanghai Slac Experimental Animal Centre. Rats were maintained in specific pathogen-free metabolic cages in a room with a 12 h light/dark cycle at an appropriate temperature of 24 ± 2 °C and relative humidity of 50 ± 5%. After acclimatizing in the metabolism cages for one week prior to dosing, the 24 rats were randomly assigned to three groups with 8 rats in each as follows: control, model and CA group. 95% (v/v) alcohol was diluted to 55% with water and 12 mL kg^−1^ was administered daily to each rat. The rats in the model group and CA group were orally administrated with ethanol at a dosage of 55% v/v once a day for 7 consecutive days to induce an animal liver injury model. The CA group was administrated with 9 mg mL^−1^ chlorogenic acid solution (7 mL kg^−1^) once a day for the following 14 days. The control group was administrated with distilled water at the same dosage. All of the experiments were approved by the Animal Care and Ethics Committee at Mudanjiang Medical University and were also performed in accordance to the declaration of Helsinki.

### 2.3 Biological sample collection and preparation

The urine samples with a volume range of approximately 5–7 mL were collected at 9:00 a.m. every day. The rats and cages were homologous during the whole experimental process to avoid any external disturbance. Then, all of the urine samples were centrifuged at 13 000 rpm, at 4 °C for 15 min and the separated supernatants were stored in liquid nitrogen until the metabolomics research. “Quality control” (QC) sample which contain a great deal of information of urine samples for optimizing LC-MS conditions were randomly choose from three groups and then put them together. At the end of the fourteen day treatment process, all of the blood samples were collected from the aorta abdominalis 24 hours after the final administration and were processed *via* centrifugation at 3000 rpm, at 4 °C for 15 min. The serum samples were transferred into Eppendorf Tubes and were stored in liquid nitrogen for clinical biochemistry examinations, conforming to standard procedures. Finally, the livers were removed from the rats promptly for hematoxylin and eosin (H&E) staining analysis.^24^

### 2.4 Clinical chemistry and histopathological examinations

Clinical biochemistry analysis was conducted using standard methods and the acquired data were analyzed using the SPSS 19.0 software. The histological morphology examinations were performed by the affiliated hospital Heilongjiang University of Chinese Medicine. H&E staining using the Image-Pro Plus 5.0 software (Media Cybernetics, Bethesda, MD, USA) for image analysis were performed on the hepatic tissues of the control, model and CA groups.

### 2.5 Metabolomic studies

#### 2.5.1 LC analysis

LC was performed on a waters system ACQUITY UPLC system (Waters Corporation, Milford, MA) conducted on an ACQUITY UPLC HSS T3 column (2.1 mm × 100 mm, 1.8 μm), which was under the control of Masslynx (V4.1, Waters Corporation, Milford, USA). The injection volume was 4 μL under a flow rate of 0.4 mL min^−1^ and the column was maintained at 45 °C. The optimal mobile phase included a linear gradient system of (A) 0.1% formic acid in acetonitrile and (B) 0.1% formic acid in water: 0–1 min, 1% A; 1–3 min, 1–11% A; 3–6 min, 11–21% A; 6–9 min, 21–40% A; 9–10 min, 99% A. All samples were maintained at 4 °C in the sample storage module. Because the QC samples contained a large amount of urine sample information, they were applied to improve the conditions for the LC-MS. The eluent was directly transferred to the mass spectrometer without spillage. A needle wash cycle was performed to wash off any remnants and be set for the next sample.

#### 2.5.2 MS analysis

MS was performed on a Waters Micromass Synapt High Definition Mass Spectrometer (Manchester, UK) equipped with an electrospray ion source in positive ion mode and negative ion mode. For the positive ion mode, the capillary voltage was set at 3.1 kV and the cone voltage at 20 V. The cone gas flow was set at 350 L h^−1^ with an ion source temperature of 300 °C. For the negative ion mode, the capillary voltage was set at 2.6 kV and the cone voltage was 20 V. The cone gas flow was set at 350 L h^−1^ with an ion source temperature of 500 °C. Data were collected in centroid mode between *m*/*z* 100 and 1000 with a 0.2 s scan time with an inter-scan delay of 0.1 s under positive and negative ion modes. All analyses were performed with an independent reference spray for data accuracy and reproducibility *via* LockSpray interference consisting of leucine enkephalin at 0.2 ng mL^−1^ infused at a flowrate of 120 mL min^−1^ which provided a reference ion for the positive ion mode ([M + H]^+^ = 556.2771) and the negative ion mode ([M − H]^−^ = 554.2614) to guarantee accuracy.

#### 2.5.3 Data processing and biomarker identification

MassLynx V4.1 software (Waters Corporation, Milford, USA) was applied to deal with the raw data from the LC-MS. Using the Apex-Track-peak detection package, MarkerLynx integrated the peaks in the LC-MS data containing the noise filtering, height intensities peak detection, removal of isotope masses and alignment of retention time (rt) and mass (*m*/*z*). PCA scores plot present original data average weights. The PCA method was applied to differentiate and assess every group status. PLS-DA and OPLS-DA score plots present accurate calculation of discrepancy among different groups of rats. PLS-DA and OPLS-DA can eliminate some variations that are not associated with the dependent variables in the independent variables. A wide range of models were applied to forecast the metabolic phenotypes and discern the different metabolites. From the PCA scores, score plots are presented indicating the scatter of the samples, which at every point in an individual sample forbade similar metabolomic ingredients when clustered together and compositionally different metabolomes when dispersed. Potential biomarkers were extracted from VIP-plots (Variable Importance for Projection-plots) generated from OPLS-DA which was applied to discover the most impactful distinction traits between the two groups. In the light of the contribution to the variation and correlation within the data set, markers were chosen for the study. Mass fragment unit manager (Waters Corp., Milford, USA) was applied to recognize the tandem mass spectrometry fragments of the metabolites. MetaboAnalyst, a web-based tool for pathway analysis and visualization metabolomics, was used to perform ingenuity pathway analysis of liver injury. Biomarkers and related biological information were analyzed using a coalescence of Metlin, HMDB and ChemSpider.^25,26^

#### 2.5.4 Statistical analyses

All analysis of the data was dealt with using the two-tailed, two-sample Student's *t*-test. Differences with a *p*-value of 0.05 or less were set down as statistically significant. Before multivariate analysis, the resultant data matrices were mean-centered and pared to scale. Studies were carried out in triplicate using data expressions with means and standard deviations (SD).

## 3. Results and discussion

### 3.1 Biochemical analysis and histopathological observations

**Fig. 1:**
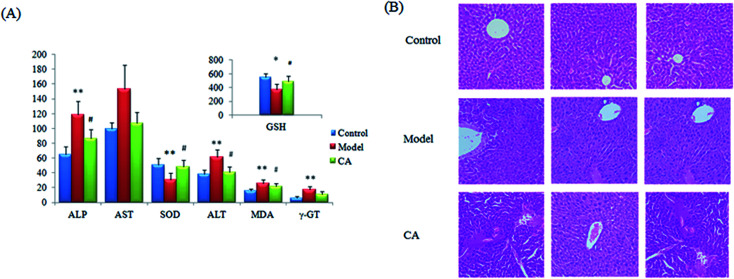
Therapeutic efficacy of chlorogenic acid against liver injury in clinical biochemistry analysis. The alcoholic liver injury resulted in significant increases in the serum levels of ALP, AST, ALT, γ-GT and MDA together with decreases in the levels for GSH and SOD compared with those of the control groups (A). Chlorogenic acid effectively reversed all of the abovementioned abnormalities, and significant differences were found in the indexes of ALP, AST, ALT, MDA, GSH and SOD compared with those of the model group. *p, 0.05; **p, 0.01; ^#^*p*, 0.05, ^##^*p*, 0.01. H&E: liver tissue from the model group showed disorderly hepatocyte cords, severe fatty degeneration, spotty or focal necrosis and infiltration of inflammatory cells; hemorrhagic necrosis with foci of lymphomonocytic infiltration around fibrosis tissue can be seen (B).

Using ethanol gavage, the alcohol-induced liver injury model was manufactured by intragastric administration with ethanol for 7 days, taking the shape of liver damage in the rats. The rats obviously presented in a drunken state, resulting in more severe in-serum concentrations of ALP, AST, ALT, γ-GT and MDA together with concentration decreases of GSH and SOD in the model rats compared with those of the control groups. GSH is an important antioxidant having the ability to scavenge free radicals *in vivo*, and the change in the number of free radicals suggests the result of a liver system injury, oxidative stress and lipid metabolism disorders. After CA effective treatment, all of the imbalances in the indicators mentioned were relieved and highlighted differences were found for most indexes except γ-GT when compared with the model samples (Fig. 1A). HE staining analysis was performed on hepatic tissue from the model, control and CA groups to examine the pathology of the liver such as cytomorphology and cellular apoptosis (Fig. 1B). The liver tissue structure from the normal group samples is normal and inconspicuous abnormalities were observed in the liver tissues such as lipid droplets, swelling and inflammatory cell infiltration. In addition, significant differences were shown in the ethology of the rats. The rats in the control group were evaluated as being in an active and normal state and rats in the model group were restless and aggressive during the initial phase and malaise and lethargic several hours later. However, compared with the model group, all of the indexes had been reversed to different levels after a 14 day treatment with the chlorogenic acid. The swollen cells, balloon-like changes and hepatic sinus congestion symptoms of the treatment group were significantly relieved. These results suggest that the alcohol-induced liver injury model was successfully established and that chlorogenic acid could ameliorate liver tissue injury caused by ethanol exposure.

### 3.2 Metabolomic analysis

The normalized data of endogenous metabolites were imported to EZinfo 2.0 software (Waters Corporation, Manchester, UK) for multivariate analysis and constructing model such as PCA, PLS and OPLS-DA. The PCA method was applied to differentiate and assess every group status. OPLS-DA or PLS-DA can eliminate some variations that are not associated with the dependent variables in the independent variables to lower the complexity of the model using parameters such as *R*^2^*Y* (cum), *Q*^2^ (cum), and *R*^2^*X* (cum) to evaluate the model quality and advance the model prediction capability. But beyond that, the OPLS-DA approach possesses the ability to clearly show the differences between the control group and the model group to discover and discriminate the biomarkers. The OPLS-DA score plots (Comp1 *R*^2^*X* (cum) = 0.905408, Comp2 *R*^2^*X* (cum) = 0.905408 in positive mode and Comp1 *R*^2^*X* (cum) = 0.999126, Comp2 *R*^2^*X* (cum) = 0.999126 in negative mode) shows obvious credible difference between the control group and the model group. Ultimately, the metabolites were intensively studied using other internet tools for screening out biomarkers.

**Fig. 2:**
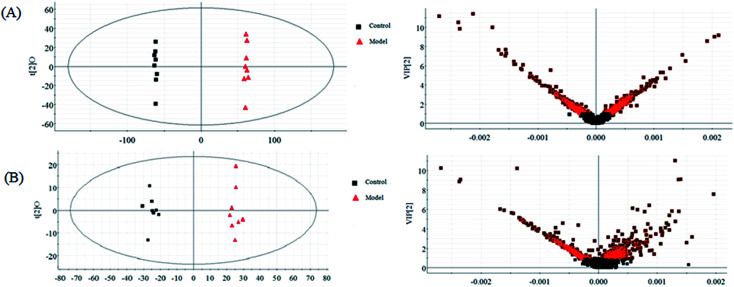
OPLS-DA and VIP score plots of the control and model groups in positive (A) and negative (B) modes.

According to the LC-MS, urine metabolomics was used to explore the potential hepatoprotective effect mechanisms of chlorogenic acid in ethanol-induced liver damage. Urine metabolic profiling was obtained using both the positive and negative ion modes of LC-MS. OPLS-DA as a high variation and prediction capability supervised method to examine LC-MS dataset discrimination between the control and model groups. From the OPLS-DA score plots (Fig. 2A and B), the model and control groups are distinctly separated, which implies that significant metabolic variations exist among these samples and that the models show pathological changes though metabolic differences resulting from pathological lesions as a vital role in the group separation. Though multivariate statistical analysis, 19 differentially expressed biomarker metabolites regarded to result in the metabolic differences between the control and model group were identified. These metabolites indicated an unbalanced metabolism arising in the liver injured animals.

**Fig. 3:**
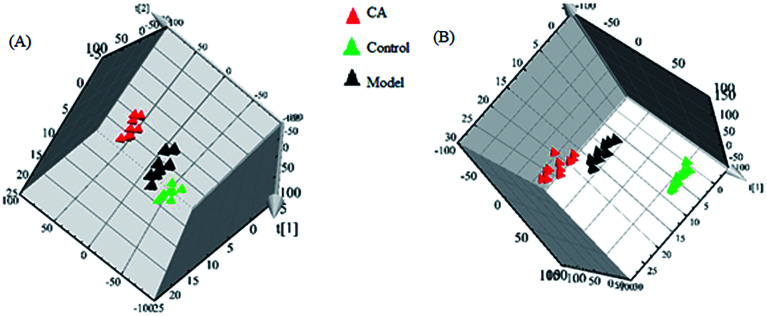
Clustering of samples from all of the different groups in positive (A) and negative (B) modes.

Typically, clustering of samples from all of the different groups is shown by the PLS-DA score plots, of which each data point represents a rat urine metabolome and the spatial distance suggests the scale of their metabolic discrepancy to some degree from the positive and negative modes. After the 14 day treatment of CA, the metabolic profiles of rats in CA group present the different tracks relative to the model group, which data point were gradually close to the normal sample state. A clear metabolic track from the in score plots suggested that the chlorogenic acid treatment showed a protective effect in reversing urinary biochemical perturbation resulting from alcohol (Fig. 3).

**Fig. 4:**
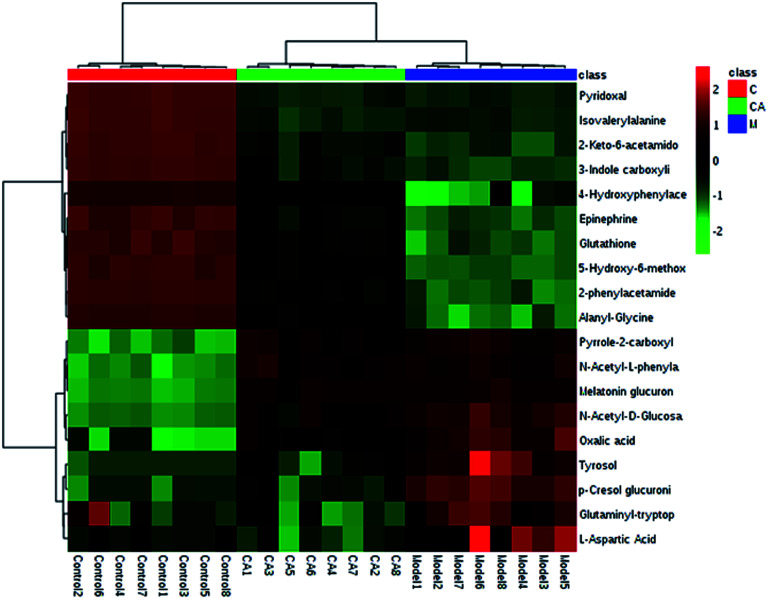
Hierarchical clustering heat map of the 19 differential metabolites with the degree of change marked with colors including up-regulation (red) and down-regulation (green).

**Fig. 5:**
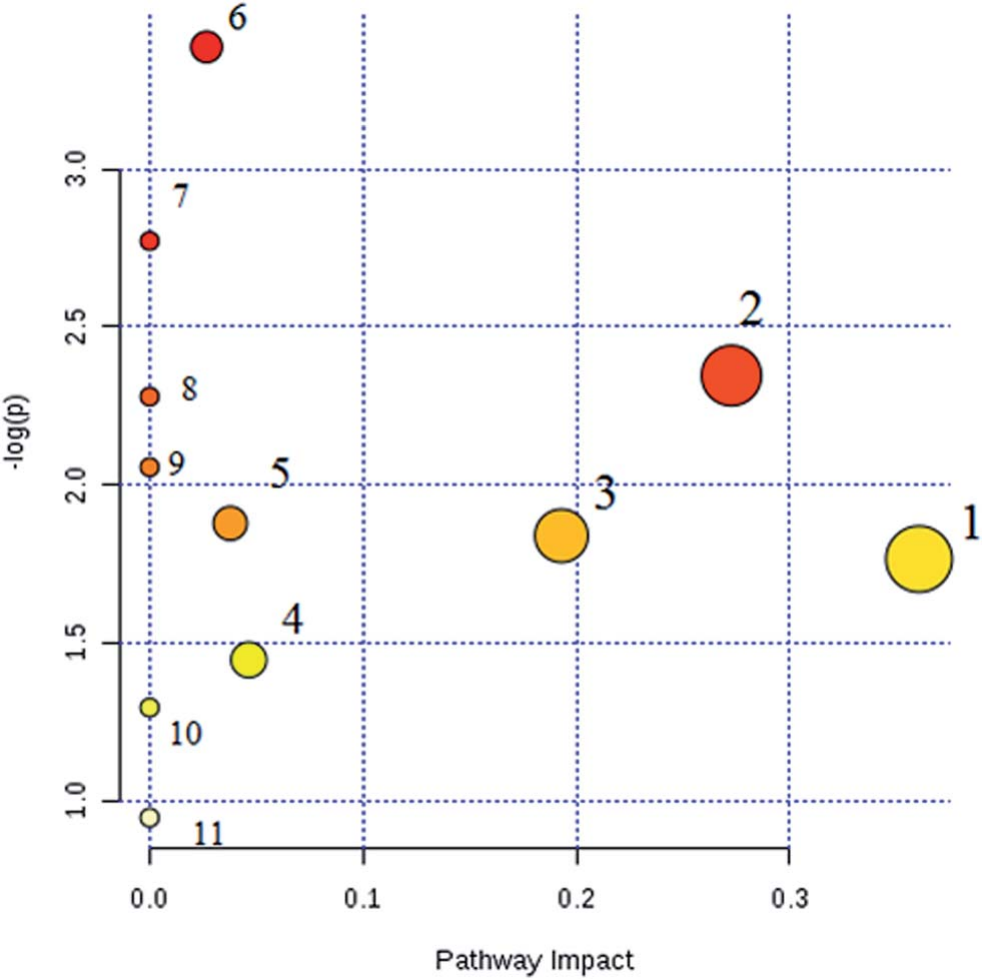
Pathway analysis of chlorogenic acid on protecting against alcohol-induced liver injury. (1) glutathione metabolism; (2) pentose and glucuronate interconversions; (3) alanine, aspartate and glutamate metabolism; (4) amino sugar and nucleotide sugar metabolism; (5) starch and sucrose metabolism; (6) tyrosine metabolism; (7) phenylalanine metabolism; (8) histidine metabolism; (9) beta-alanine metabolism; (10) arginine and proline metabolism; (11) aminoacyl-tRNA biosynthesis.

Interrelation of the correlation matrix among the 19 biomarkers using Pearson's linear correlation analysis with different degrees of change in the control, CA and model groups are shown in Fig. 4. The color scale shows the magnitude and direction of the relationship of the 19 biomarkers among the different groups, with red representing the positive and green representing the negative correlations. We identified a total of 14 distinct metabolic biomarkers after CA treatment including glutaminyl-tryptophan, glutathione, 4-hydroxyphenylacetaldehyde, 2-phenylacetamide, epinephrine, l-aspartic acid, *N*-acetyl-d-glucosamine 6-phosphate, alanyl-glycine, 2-keto-6-acetamidocaproate, oxalic acid, tyrosol, 3-indole carboxylic acid glucuronide, *p*-cresol glucuronide and 5-hydroxy-6-methoxyindole glucuronide. Then, pathway analysis related with pharmacological action of anti-liver injury of CA was based on the MetaboAnalyst software which is a powerful way to probe into the most relevant pathways associated with in a specific condition (Fig. 5).

## 4. Discussion

High-throughput metabolomics represents a powerful approach for revealing new mechanistic insights into natural products.^27–30^ The liver is the most vital organ in bio-metabolism offering basic metabolic, exocrine and endocrine functions. Metabolic disorders resulting from liver disease are often extensive. Alcohol-induced liver injury is mainly through the following pathway: NADH refers to the coenzyme of fatty acid synthesis and NAD is the coenzyme of fatty acid oxidation. NADH/NAD imbalance leads to increased synthesis and reduced decomposition of fatty acids, which results in a large number of triglycerides in the liver associated with a fatty liver;^31^ the toxic effects of ethanol and acetaldehyde on hepatocytes cause cell membrane damage; this then causes antigen changes in the liver cells due to their own immune response; ethanol hypercholesterolemia causes hepatocyte hypoxia; acetaldehyde promotes collagen synthesis, which is related to inflammation stimulating collagen fiber proliferation to cause liver fibrosis.^32^ From clinical biochemical indicators, toxic reactions, abnormal amino acid metabolism and oxidation–reduction reaction were emerged in the rat body. The alcoholic liver injury results in significant increases in the serum levels of ALP, AST, ALT, γ-GT and MDA together with decreases in the GSH and SOD levels compared with those of the control groups. According to histopathological observations of the liver, the liver injury model of the rats was also proven to be successful.

CA reverses the above abnormalities in an effective way. Metabolomics studies can detect the significant alteration of various endogenous metabolites. CA notably regulates 14 metabolites and 11 metabolic pathways including glutathione metabolism, pentose and gluconate metabolism, as well as amino acid metabolism, to have a protective effect on ethanol-induced liver injury. Glutathione, as a natural cell antioxidant, composed of glutamic acid, cysteine and glycine, is rich in the liver and is characterized by antioxidant activity and detoxification.^33^ When the liver is damaged, such as suffering from a variety of liver diseases, the body will provide a lot of glutathione for self-rehabilitation and detoxification. From the biochemical indicators, the reduction of glutathione content *in vivo* due to glutathione peptide peroxidase depletion and decreased activity is one of the gold indicators of liver cell damage in the clinic. The liver is the largest detoxification organ in the body. Toxic substances entering into the blood by various ways are converted into water-soluble substances and other non-toxic or low toxicity compounds mostly by liver cell metabolism and are then excreted though the kidney or bile.^34,35^ One of the liver detoxification methods is the target binding effect by oxidation, reduction, hydrolysis and other ways to work on toxic chemical groups and to change their physical and chemical properties, such as molecular size, as well as their water solubility. Glucuronide detoxification plays an important role in the process of combination detoxification. The liver is an important place for amino acid and carbohydrate synthesis and metabolism. It was reported that tyrosinemia, hepatocellular disease, fatty infiltrating hepatocytes, necrosis and jaundice are related to the abnormal metabolism of amino acid enzymes. The pathways associated with these biomarkers indicate their significant disorder in ethanol-induced liver injury which may lead to disease progression.

## 5. Conclusion

The liver is not only the major organ for ethanol metabolism, but also the primary objective organ for alcohol consumption. In recent years, studies on the protective mechanisms of active components from natural products for protection against liver injury by alcohol intake have attracted more and more attention. Using metabolism analysis based on metabolomic technologies, the therapeutic effect of CA on ethanol-induced liver injury has been revealed and the pivotal live protection molecular mechanisms were probed. The results suggested that CA remarkably reversed ethanol-induced liver injury by rebalancing the glutathione metabolism, carbohydrate metabolism and multiple amino acid metabolisms. Although the pathogenesis of ethanol-induced liver injury is extremely complicated and the target objective of CA is also broad, this study based on metabolomics brings about a better understanding of the action mechanisms of CA on ethanol-induced liver injury.

## Conflicts of interest

There are no conflicts to declare.

## Supplementary Material
